# Commitment devices in the treatment of diabetic foot ulcers

**DOI:** 10.1186/s13047-019-0355-9

**Published:** 2019-08-19

**Authors:** Gustav Jarl

**Affiliations:** 1 Department of Prosthetics and Orthotics, Faculty of Medicine and Health, Örebro University, SE 70182 Örebro, Sweden; 2 University Health Care Research Center, Faculty of Medicine and Health, Örebro University, SE 70182 Örebro, Sweden

**Keywords:** Diabetes complications, Diabetic foot, Shoes, Patient compliance, Treatment adherence and compliance, Casts, surgical, Shoes, Orthotic devices

## Abstract

**Background:**

Non-removable offloading devices are recommended for the treatment of uncomplicated plantar diabetic foot ulcers because adherence to using removable devices is low. However, patients may not always understand how crucial the non-removability is to ulcer healing, leaving them with the impression that it is the device per se that heals the ulcer. Thus, after ulcer healing when patients return to using removable offloading devices, typically therapeutic footwear, they often return to a low level of adherence resulting in high reulceration rates. To change this pattern of behavior based on a misconception, we need to start with how we as clinicians are conceptualizing treatment with offloading devices.

**Non-removable offloading devices as commitment devices:**

Commitment devices are voluntary restrictions people put on their future selves to resist short-term temptations and achieve long-term goals. In this paper, it is suggested that a change from viewing non-removable offloading devices as means to force compliance, to viewing them as commitment devices could facilitate a change to a clinical thinking that emphasizes the importance of high adherence without compromising respect for patient autonomy.

**Conclusion:**

Viewing non-removable offloading devices as commitment devices seems to be a promising approach to emphasize the importance of adherence while respecting patient autonomy. Hopefully, patients’ higher appreciation of the role of adherence can lead to higher adherence to using therapeutic footwear after healing and consequently to reduced reulceration rates.

## Background

Diabetic foot ulcers are a common and devastating complication of diabetes, and are associated with significant morbidity, mortality, and risk of amputation [1]. Non-removable offloading devices are recommended in the treatment of uncomplicated plantar foot ulcers [2] because adherence to using removable offloading devices is often low [3, 4]. Once the ulcer is healed, patients change to using removable offloading devices, typically therapeutic footwear, to mitigate plantar pressures and prevent reulceration [5]. One may think that the experience of effective healing when using a non-removable device would convince patients of the importance of adherence, resulting in high adherence to wearing therapeutic footwear after healing and thus ensuring low reulceration rates. Unfortunately, many patients do not seem to acknowledge that the non-removability of the device ensures high adherence to using it, which allows the ulcer to heal; instead, they attribute the entire healing outcome to the device’s offloading effect, that is, the ability of the device to reduce mechanical stresses on the ulcer. In one study [6], patients were interviewed about their experiences of using total contact casts and removable walkers. Interestingly, they were aware of the more effective ulcer healing when using total contact casts but attributed it – falsely – to casts offloading the ulcer more effectively than walkers do; they did not attribute better healing to the non-removability of casts resulting in higher adherence. This stands in stark contrast to research results, which demonstrate that walkers can offload forefoot ulcers equally effectively as casts [7, 8] and that walkers provide similar healing outcomes as casts if the walkers are rendered non-removable [6]. Patients’ underestimation of the importance of adherence may be one of the reasons for low adherence to wearing therapeutic footwear after ulcer healing [9] and for the high reulceration rates: approximately 40% of patients develop a new ulcer within the first year after healing [1].

One may speculate that the way clinicians frame and present treatment with non-removable offloading devices may not communicate to patients the importance of high adherence. All too often, the clinical focus is on the device itself, leaving patients with the impression that it is the device per se that heals the ulcer. In reality, healing of uncomplicated plantar ulcers is mainly determined by two factors combined: effective offloading of the ulcer and high adherence to using the offloading device. Hence, non-removable devices have been proposed as a way to force compliance and thereby reach the desired level of adherence [4, 10]. However, the concept of non-removable devices as a means to force compliance has a paternalistic connotation which is not compatible with viewing patients as partners in decision-making. Furthermore, it conveys the picture of clinicians as active and patients as passive in the decision. Although unintended, these connotations may counteract the sense of long-term personal responsibility for adherence that is crucial after healing, when removable devices are used to prevent reulceration. Thus, an alternative way to conceptualize treatment with non-removable offloading devices is needed.

## Non-removable offloading devices as commitment devices

**Fig. 1:**
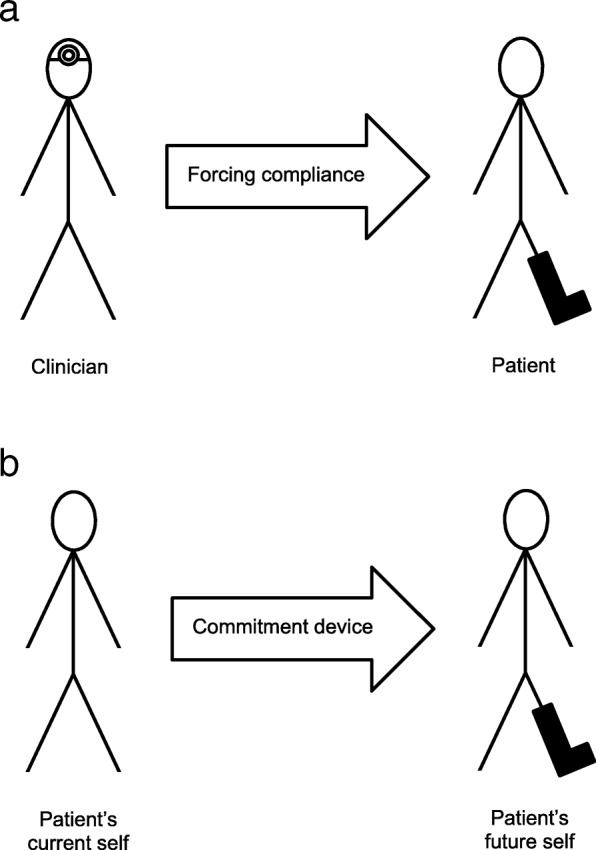
Non-removable offloading devices conceptualized in two ways: **a**. as a means to force compliance: the clinician (active partner) prescribes a non-removable device to force the patient (passive partner) to be adherent in using the device. **b**. as commitment devices: the patient’s current self commits to using a non-removable device to prevent his or her future self from being non-adherent in using the device

Commitment devices are voluntary restrictions that people put on their future selves to resist short-term temptations and achieve long-term goals [11]. For example, a person may undergo gastric bypass surgery to guard against future temptations to overeat, and thereby reduce calorie intake and lose weight. This line of thinking fits well in the context of adherence to using offloading devices; the long-term goal is to heal a foot ulcer and the short-term temptation is to engage in “strategic non-adherence”, that is, purposely being non-adherent in an attempt to live a normal life [12, 13].The solution is to use a non-removable device, which in this sense could be labeled a commitment device; the patient agrees to use the device and commits to using it continuously, even when his or her future self is tempted to remove it. In contrast to viewing non-removable devices as a means to force compliance on a passive patient, the patient’s current self can be invited to take an active decision to restrict the opportunities for his or her future self to be non-adherent (Fig. 1).

Hence, the importance of adherence is made salient without compromising respect for the patient’s autonomy and self-determination. In addition, conceptualizing non-removable devices as commitment devices highlights the need to discuss the gap between intentions and future behaviors with patients [11], a discussion that should precede the transition from using a non-removable device during treatment to using removable offloading devices after healing. Now the focus is on educating the patient about the effectiveness of the offloading device, typically therapeutic footwear, and establishing long-term habits of high adherence. The literature suggests different approaches to strengthen the patient’s motivation for adherence and to bridge the intention–action gap, but the evidence is in most cases weak and more research is needed. For example, patients may be educated about the offloading effect by measuring and visualizing plantar pressures [14], and adherence may be improved with motivational interviewing [15] and other structured communication techniques, such as person-centered communication and shared decision-making [16]. Furthermore, patients can be advised to keep their therapeutic footwear visible at home to provide a cue to use it and to put their conventional shoes away to eliminate the temptation to use them [17]. Hopefully, by emphasizing the importance of adherence, ensuring that the patient is an active agent during ulcer treatment, and addressing the intention–action gap, higher adherence to removable offloading devices after healing can be achieved.

Although commitment devices per se have not been discussed previously in the context of offloading diabetic foot ulcers, the underlying principle of self-imposed restrictions is already at work in the field. A study in which people with foot ulcers wore non-removable therapeutic footwear illustrates this [18]. In the questionnaire asking participants for perceived advantages and disadvantages with the treatment regimen, one participant reported that an advantage of non-removable footwear was that he was not tempted to walk without his therapeutic footwear. This illustrates the idea of viewing non-removable offloading devices as commitment devices; the idea is not to force compliance on patients but to suggest an effective, still voluntary, means to improve adherence through a self-imposed restriction on everyday choices available to the person’s future self.

How we choose to name things influences how we perceive them and, by extension, how we act in clinical practice. Thus, it is important to choose and use concepts that provide an appropriate clinical mindset when meeting our patients. For example, in one study, the term “*diabetic foot attack*” was introduced to emphasize the urgency of certain clinical presentations of diabetic foot disease, such as an acutely inflamed foot with rapidly progressing tissue necrosis [19]. Another study proposed the “*in remission*” concept as an alternative to viewing patients as being cured, in order to prepare patients and clinicians for inevitable future complications and to emphasize the need for frequent follow-up [20]. A third study suggested the “*process perspective*” to highlight diabetic foot disease as a single process consisting of both active (healing) and latent (prevention) phases, which could be a more fruitful model for understanding inadequate patient behaviors that are not easily understood from a dichotomous healing–prevention perspective [21]. Hopefully, conceptualizing non-removable offloading devices as commitment devices to aid adherence could be a part of this expanding conceptual framework to support clinicians in their work and their communication with patients.

## Conclusions

Viewing non-removable offloading devices as commitment devices seems to be a promising approach to emphasize the importance of adherence without compromising respect for patient autonomy. Hopefully, this will result in higher adherence to using removable offloading devices after healing, which would in turn lower reulceration rates. Other researchers are invited to elaborate on this concept and investigate its practical implications.

## Data Availability

Not applicable.
